# A 10-year retrospective repeated cross-sectional study of occupational radiation exposure trends among radiology staff in two tertiary hospitals in Chongqing

**DOI:** 10.3389/fpubh.2026.1888215

**Published:** 2026-08-05

**Authors:** Yeyao Wu, Mengyun Wu, Jinghua Zhou, Nan Jin, Jing Jin, Fang Yuan, Wei Li

**Affiliations:** Radiology Laboratory, Department of Occupational Health and Radiological Health, Chongqing Center for Disease Control and Prevention, Chongqing, China

**Keywords:** hospital comparison, interventional radiology, occupational exposure, radiation dose monitoring, radiology staff, temporal trends, tertiary hospitals

## Abstract

**Background:**

This study aimed to investigate 10-year trends in occupational radiation exposure among radiology staff in two tertiary hospitals in Chongqing, China, and to compare exposure patterns across occupational categories, with particular attention to interventional radiology personnel.

**Methods:**

A repeated cross-sectional study was conducted using annual dosimetry records from radiation workers at two tertiary hospitals (an urban cancer center and a suburban general hospital) from 2015 to 2025. Annual effective doses were measured using thermoluminescent dosimeters. Data were analyzed for overall dose levels, temporal trends, high-dose events (>5, >10, >20 mSv), and occupational differences. Linear regression was used to evaluate temporal trends.

**Results:**

The number of radiation workers increased significantly in both hospitals (both *p* < 0.001). Overall mean annual doses ranged from 0.24 to 0.69 mSv, well below the national limit of 20 mSv. Interventional radiology staff consistently had the highest mean doses among all categories in both hospitals. However, the highest maximum doses were from radiotherapy staff in the urban cancer center (45.42 mSv in 2017). The suburban general hospital showed higher dose variability, more frequent high-dose events (19 vs. 5 records), and a significant increasing trend in interventional radiology mean doses (*β* = 0.08 mSv/year, 95% CI: 0.04–0.12, *p* = 0.003), whereas the urban cancer center maintained stable low doses with no significant temporal trend. These inter-hospital differences reflect a combination of hospital type, service mix, and management practices, and should not be attributed solely to urban-suburban location.

**Conclusion:**

Occupational radiation doses in both hospitals were generally within safe limits. Interventional radiologists remain the highest-risk group by mean dose, while extreme outliers were observed in radiotherapy. The two hospitals showed different dose stability and high-dose event patterns, highlighting the need for targeted protection strategies tailored to each institution’s occupational structure and risk profile.

## 1 Introduction

Many studies have confirmed that long-term exposure to low-dose ionizing radiation can damage human health. Epidemiological evidence shows that radiation workers have a significantly increased risk of cataract, thyroid nodules, hematological diseases, and cancer due to long-term occupational contact with low-dose radiation (1–3). For example, Publication 118 of the International Commission on Radiological Protection states that the lens of the eye is more sensitive to radiation than previously recognized, and a cumulative dose exceeding 0.5 gray (Gy) may induce posterior subcapsular cataract (4). In interventional radiology procedures, physicians work very close to X-ray sources. The radiation dose to the skin of the hands can reach several millisieverts (mSv) during a single procedure, and repeated exposure may lead to radiation-induced skin injury (5, 6). Therefore, protecting the health of radiation workers is critically important. It concerns not only individual safety but also the sustainable provision of radiological diagnosis and treatment services in medical institutions.

Personal dose monitoring is widely recognized as the gold standard in radiation protection. The Law of the People’s Republic of China on the Prevention and Control of Occupational Diseases and the Measures for the Administration of Occupational Health of Radiation Workers clearly require that employers carry out personal dose monitoring for all radiation workers and establish lifelong dose records. Dose monitoring serves multiple purposes: identifying high-exposure jobs, evaluating the effectiveness of protective measures, providing legal evidence for the diagnosis of occupational diseases, and verifying compliance with the principle of as low as reasonably achievable (7). Without accurate dose data, any improvement in radiation protection lacks a scientific basis.

Interventional radiology (including cardiovascular intervention and neurointervention) and nuclear medicine are high-risk occupational categories. Staff in these fields stay near X-ray equipment for long time (8, 9). Because interventional operating rooms require sterile conditions, additional shielding such as lead screens often cannot be used. As a result, radiation doses to the trunk and hands are much higher than those in conventional diagnostic radiology. Multicenter surveys show that the median annual effective dose for interventional radiologists is 2 to 5 mSv, and the dose for high-workload operators may exceed 10 mSv. The equivalent dose to the hands may approach or even exceed the occupational limit of 20 mSv per year (10, 11). In contrast, staff performing routine computed tomography and digital radiography usually stay away from radiation sources and use fixed protective facilities. Their average annual effective dose is mostly below 1 millisievert (12, 13). Recent systematic reviews have synthesized the available evidence on occupational radiation exposure among healthcare professionals, confirming that interventional staff consistently receive the highest doses across all radiology specialties (1). A study from Turkey evaluating interventional cardiology staff found that procedure doses and staff attitudes toward radiation safety varied considerably, highlighting the importance of safety culture and consistent protective practices (14).

However, most existing data on occupational radiation exposure come from developed regions or large urban hospitals. Even within the same city, tertiary hospitals may differ substantially in equipment conditions, human resources, and management standards. Whether these differences lead to varying levels of radiation exposure among staff remains unclear, and well-designed comparative studies are still lacking. The limited available evidence suggests that hospital-level factors—including equipment renewal cycles, staffing levels, and safety culture—may influence occupational dose profiles, but few studies have systematically compared dose patterns across different types of tertiary hospitals within the same geographic region.

Chongqing, a municipality in western China with concentrated medical resources, presents unique geographic and healthcare characteristics. Some tertiary hospitals benefit from timely equipment updates, high patient volumes, and strong regulatory oversight, whereas others face challenges such as aging equipment, insufficient radiation protection staff, and limited training opportunities. These contextual factors may influence occupational dose profiles, but their effects have not been systematically examined.

Against this background, we focused on two affiliated hospitals of Chongqing University. Chongqing University Cancer Hospital is a Grade A tertiary cancer center integrating medical treatment, teaching, and scientific research. Chongqing University Fuling Hospital is the largest Grade A tertiary general hospital in Fuling District, located approximately 120 km east of downtown Chongqing. It is important to note that these two hospitals differ not only in geographic location but also in specialty focus (cancer center vs. general hospital) and service composition, which may independently influence patient case mix, procedure types, workload, and equipment utilization.

In this study, we analyzed personal dose monitoring data of radiation workers at these two hospitals from 2015 to 2025. We compared overall dose levels, exposure differences among occupational groups (including interventional radiology, nuclear medicine, diagnostic radiology, and radiotherapy), annual temporal trends, and the frequency of high-dose events (annual effective dose >5 mSv). We paid particular attention to interventional radiology personnel, who are recognized as a high-risk group. Through these analyses, we aimed to: (1) characterize the 10-year occupational radiation exposure trends in two distinct tertiary hospitals; (2) identify which occupational categories are most exposed; (3) compare dose patterns between the two institutions on a like-for-like basis within the same occupational categories; and (4) provide evidence for optimizing radiation protection strategies tailored to different hospital settings.

## 2 Methods

### 2.1 Study design and population

A repeated cross-sectional study using retrospective dosimetry records was performed including all radiation workers with at least one valid annual dosimetry record at two tertiary hospitals between 1 January 2015 and 31 December 2025. Workers were excluded if dose records were incomplete, missing or covered <6 months in a given year. A total of 51 records were excluded across the study period (39 from Cancer Hospital and 12 from Fuling Hospital). As the data were derived from annual summaries of institutional dosimetry archives rather than individual-level longitudinal tracking of the same workers over time, the design is more appropriately characterized as a repeated cross-sectional study. The two hospitals were Chongqing University Cancer Hospital (urban tertiary cancer center) and Chongqing University Fuling Hospital (suburban regional tertiary general hospital), approximately 120 km apart.

### 2.2 Data collection

Personal annual effective doses (mSv) were obtained from routine institutional dosimetry archives. Thermoluminescent dosimeters were worn at the left chest/shoulder outside the lead apron and replaced quarterly. Annual effective dose was the sum of four quarterly values. The detection limit was 0.05 mSv, which was determined by the dosimeter specifications (LiF: Mg, Cu, P TLD-100H) and the laboratory’s calibration procedures in accordance with the Chinese national standard GBZ 128–2019. Values below this threshold were recorded as 0.05 mSv. This substitution may introduce a slight upward bias in the estimation of mean, median, and percentile doses, particularly in years with a high proportion of non-detectable readings. However, since the vast majority of readings were above the detection limit in both hospitals (>95% of all quarterly records), the impact on annual summary statistics is expected to be minimal. Collected variables included year, hospital, sex, occupational category and annual effective dose (mean, median, 95th percentile (P95), maximum). Occupational categories included interventional radiology, diagnostic radiology, radiotherapy, nuclear medicine, dental radiology and research radiology.

### 2.3 Statistical analysis

Data was analyzed using GraphPad Prism 8.0. Continuous variables were summarized as mean, median, P95 and maximum. Categorical variables were presented as counts and percentages. (1) the total number of radiation workers (workforce growth) for each hospital separately; (2) overall mean annual doses for all radiation workers for each hospital separately; and (3) mean annual doses for the interventional radiology subgroup for each hospital separately. Separate regressions were run for each hospital to allow direct comparison of trends between the two institutions. For workforce growth, we also compared the regression slopes between the two hospitals using a test for interaction. All regression results are summarized in Table 1. High-dose events were defined as annual effective doses >5 mSv, >10 mSv and >20 mSv. A two-sided *p*-value <0.05 was considered statistically significant.

**Table 1:** Summary of linear regression results for temporal trends in workforce size, overall mean doses, and interventional radiology mean doses.

Analysis/subgroup	Hospital	Outcome variable	*β* coefficient	Standard error	95% CI	*R* ^2^	*t* statistic	Exact *P*-value	Brief interpretation
Workforce growth	Cancer	Number of radiation workers	44.77/year	3.72	36.68 to 52.86	0.94	12.03	<0.001	Significant increase
Workforce growth	Fuling	Number of radiation workers	16.84/year	1.38	13.90 to 19.78	0.96	12.17	<0.001	Significant increase
Overall mean dose	Cancer	Annual mean dose (mSv)	−0.002/year	0.018	−0.042 to 0.038	0.01	−0.11	0.91	No significant trend
Overall mean dose	Fuling	Annual mean dose (mSv)	0.027/year	0.012	0.001 to 0.053	0.35	2.21	0.055	Borderline increasing trend
IR mean dose	Cancer	IR annual mean dose (mSv)	−0.02/year	0.025	−0.08 to 0.04	0.12	−0.81	0.44	No significant trend
IR mean dose	Fuling	IR annual mean dose (mSv)	0.08/year	0.019	0.04 to 0.12	0.67	4.12	0.003	Significant increasing trend

## 3 Results

### 3.1 Study population and data quality

A total of 4,249 annual dosimetry records from radiation workers at the two hospitals were reviewed. After excluding 51 records (39 from Cancer Hospital and 12 from Fuling Hospital) with incomplete data or monitoring periods of less than 6 months, 4,198 records were included in the final analysis. The numbers of included, excluded, and total records for each hospital and year are presented in Supplementary Table S1. The exclusion rates were low in both hospitals (1.3% for Cancer Hospital and 0.8% for Fuling Hospital), indicating high data completeness and good compliance with monitoring requirements.

### 3.2 Hospital characteristics and workforce composition

The two hospitals differed in their institutional type and workforce structure. Chongqing University Cancer Hospital is an urban tertiary cancer center, while Chongqing University Fuling Hospital is a suburban regional tertiary general hospital.

**Figure 1:**
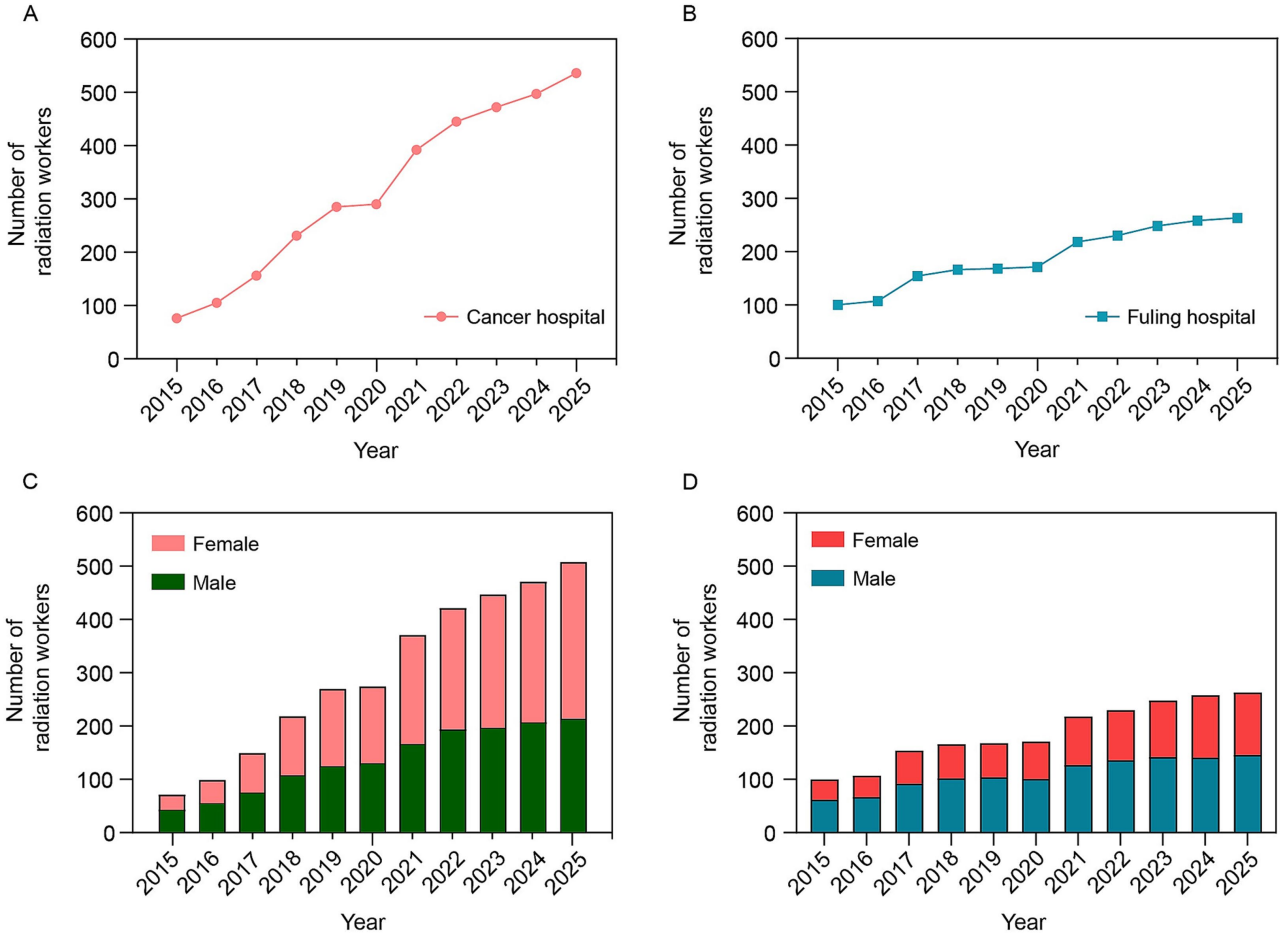
Number and sex distribution of radiation workers in two tertiary hospitals, 2015–2025. **(A)** Total number of radiation workers at Chongqing University Cancer Hospital. **(B)** Total number of radiation workers at Chongqing University Fuling Hospital. **(C)** Sex distribution at Cancer Hospital (pink: female, green: male). **(D)** Sex distribution at Fuling Hospital (red: female, blue: male).

Between 2015 and 2025, the number of monitored radiation workers increased substantially in both hospitals (Figures 1A,B and Table 2). Linear regression analysis confirmed a significant upward trend in workforce size at both hospitals (Table 1; Cancer Hospital: *β* = 44.77 workers/year, 95% CI: 36.68–52.86, *p* < 0.001; Fuling Hospital: *β* = 16.84 workers/year, 95% CI: 13.90–19.78, *p* < 0.001). The growth slope at Cancer Hospital was significantly steeper than that at Fuling Hospital, as indicated by the non-overlapping 95% confidence intervals (test for interaction, *p* < 0.05). At Cancer Hospital, the workforce grew from 76 to 536 (a 7.1-fold increase), and at Fuling Hospital, it grew from 100 to 263 (a 2.6-fold increase). The proportion of female workers rose from 39.47 to 57.84% at Cancer Hospital and from 38.00 to 44.49% at Fuling Hospital (Figures 1C,D).

**Table 2:** Number and sex distribution of radiation workers in two tertiary hospitals, 2015–2025.

Year	Chongqing university cancer hospital				Chongqing university fuling hospital			
	Total	Male	Female	Female (%)	Total	Male	Female	Female (%)
2015	76	46	30	39.47	100	62	38	38.00
2016	105	59	46	43.81	107	67	40	37.38
2017	158	80	78	49.37	154	92	62	40.26
2018	231	114	117	50.65	166	102	64	38.55
2019	285	132	153	53.68	168	104	64	38.10
2020	290	138	152	52.41	171	101	70	40.94
2021	392	176	216	55.10	218	127	91	41.74
2022	445	205	240	53.93	230	136	94	40.87
2023	472	208	264	55.93	248	142	106	42.74
2024	497	219	278	55.94	258	141	117	45.35
2025	536	226	310	57.84	263	146	117	44.49

**Figure 2:**
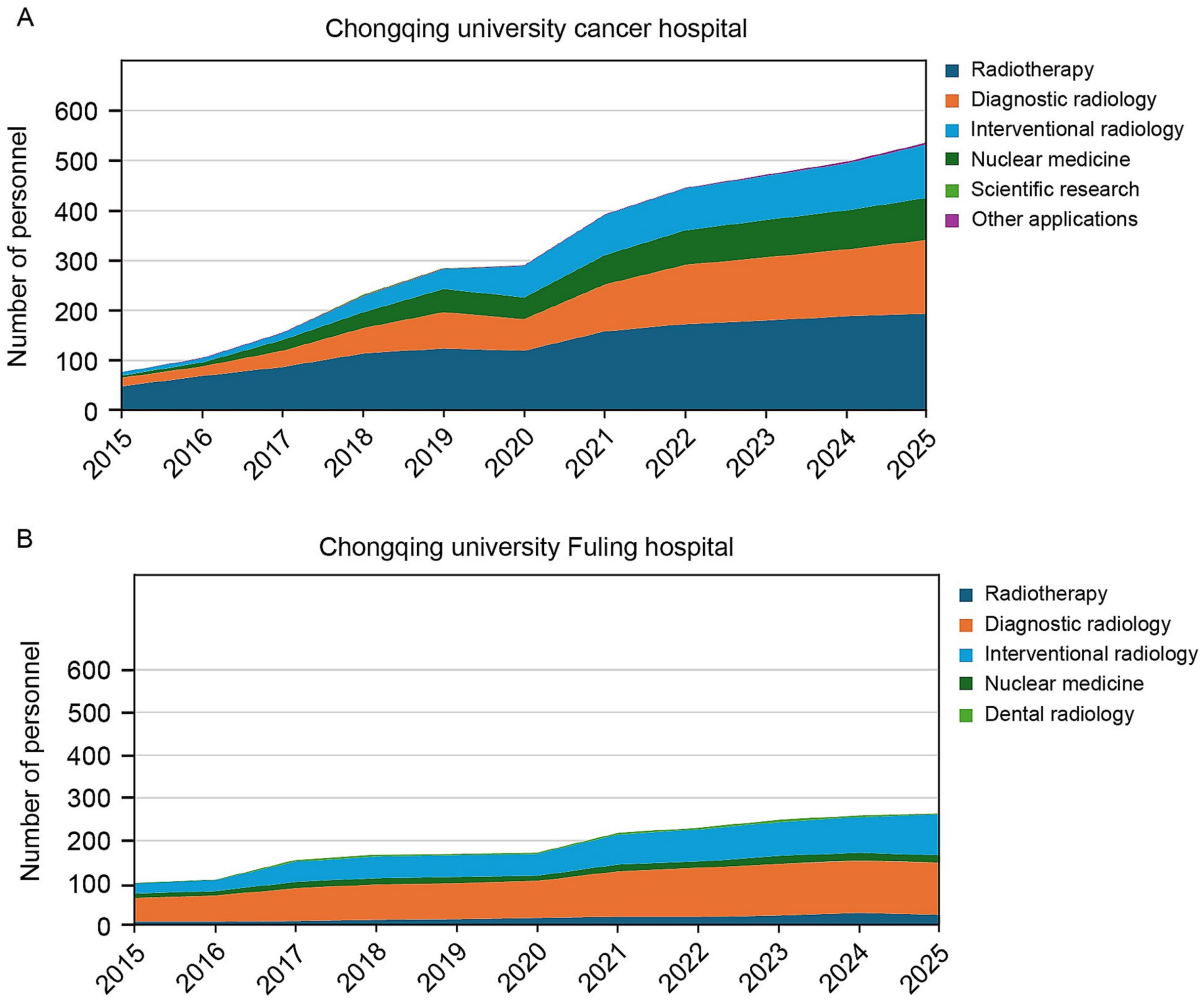
Annual distribution of radiation workers by occupational category, 2015–2025. **(A)** Staff distribution by specialty at Chongqing University Cancer Hospital. **(B)** Staff distribution by specialty at Chongqing University Fuling Hospital.

The distribution of workers across occupational categories also differed between the two institutions (Figures 2A,B and Table 3). At Cancer Hospital, the workforce was more diversified, with notable growth in radiotherapy (48–193), nuclear medicine (5–84), and especially interventional radiology (6–108, a 17-fold increase). At Fuling Hospital, diagnostic radiology consistently accounted for the largest group (55–122), while interventional radiology staff increased from 25 to 96. Radiotherapy and nuclear medicine remained relatively small categories at Fuling Hospital.

**Table 3:** Distribution of radiation workers by occupational category in two tertiary hospitals, 2015–2025.

Year	Hospital	Occupational category						
		Radiotherapy	Diagnostic radiology	Interventional radiology	Nuclear medicine	Scientific research	Dental radiology	Other applications
2015	Cancer	48	17	6	5	0	0	0
	Fuling	10	55	25	9	0	1	
2016	Cancer	68	20	8	8	0	0	1
	Fuling	10	61	25	10	0	1	
2017	Cancer	87	33	14	21	0	0	1
	Fuling	11	77	48	14	0	4	
2018	Cancer	114	51	32	31	2	0	1
	Fuling	14	83	51	14	0	4	
2019	Cancer	124	72	39	47	2	0	1
	Fuling	15	85	50	14	0	4	
2020	Cancer	119	64	63	43	0	0	1
	Fuling	17	88	51	12	0	3	
2021	Cancer	158	94	80	59	0	0	1
	Fuling	20	108	70	15	0	5	
2022	Cancer	173	118	83	70	0	0	1
	Fuling	20	115	75	15	0	5	
2023	Cancer	180	126	88	75	0	0	3
	Fuling	24	121	80	18	0	5	
2024	Cancer	188	134	94	78	0	0	3
	Fuling	29	123	84	19	0	3	
2025	Cancer	193	148	108	84	0	0	3
	Fuling	25	122	96	18	0	2	

### 3.3 Overall annual dose levels and temporal trends

**Figure 3:**
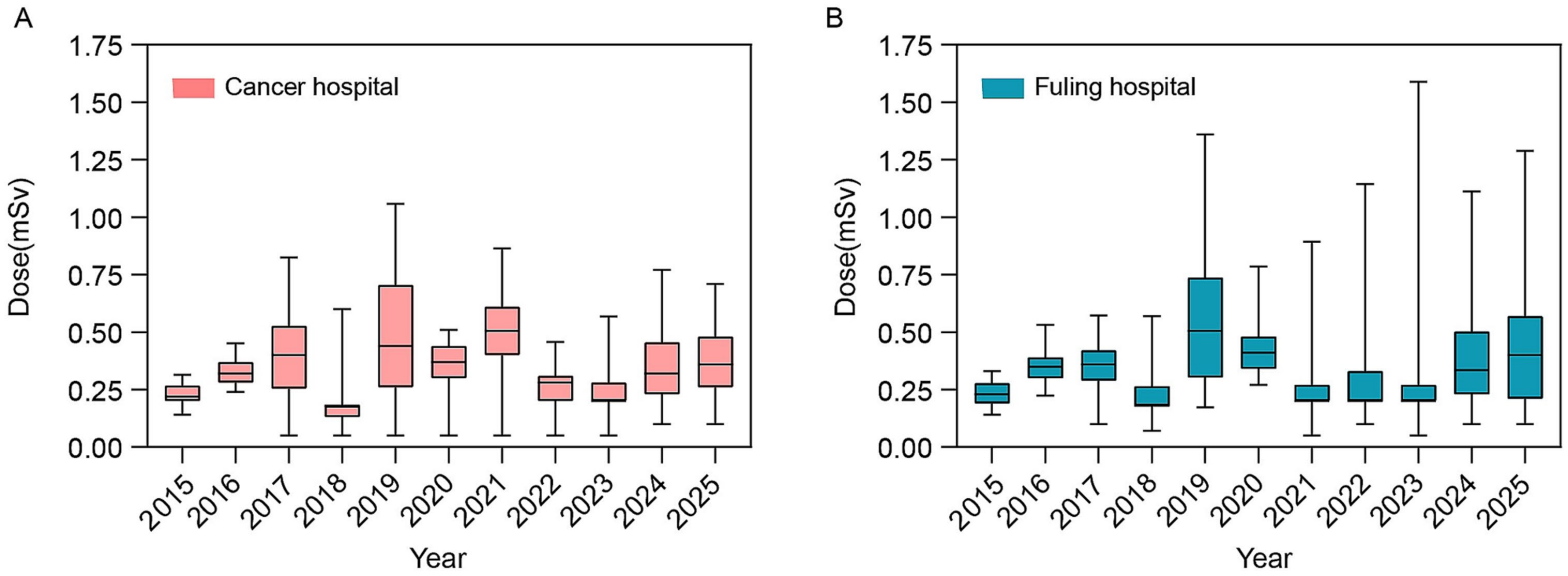
Annual effective dose distribution among radiation workers, 2015–2025. **(A)** Annual distribution of individual annual effective doses (mSv) among radiation workers at Chongqing University Cancer Hospital. **(B)** Annual distribution of individual annual effective doses (mSv) among radiation workers at Chongqing University Fuling Hospital. The box represents the interquartile range (25th–75th percentiles), the line inside the box indicates the median, and the whiskers extend to the 5th and 95th percentiles.

Overall, annual effective doses for all radiation workers remained well below the Chinese national occupational limit of 20 mSv in both hospitals (Figure 3 and Table 4).

**Table 4:** Annual effective dose among radiation workers in two tertiary hospitals, 2015–2025.

Year	Chongqing university cancer hospital					Chongqing university Fuling hospital				
	*N*	Mean (mSv)	Median (mSv)	P95 (mSv)	Max (mSv)	*N*	Mean (mSv)	Median (mSv)	P95(mSv)	Max (mSv)
2015	76	0.24	0.22	0.31	0.95	100	0.24	0.23	0.33	0.82
2016	105	0.35	0.32	0.43	1.21	107	0.38	0.35	0.51	1.78
2017	156	0.69	0.40	0.78	45.42	154	0.37	0.36	0.55	2.21
2018	231	0.30	0.18	0.60	14.11	166	0.24	0.18	0.56	1.21
2019	285	0.52	0.44	1.02	3.08	168	0.60	0.51	1.29	3.49
2020	290	0.36	0.37	0.51	0.79	171	0.52	0.41	0.74	11.23
2021	392	0.62	0.51	0.85	16.39	218	0.37	0.20	0.73	17.00
2022	445	0.28	0.28	0.45	1.67	230	0.42	0.20	1.11	8.77
2023	472	0.31	0.20	0.51	19.97	248	0.68	0.20	1.16	32.13
2024	497	0.39	0.32	0.76	7.12	258	0.54	0.34	1.10	13.59
2025	536	0.38	0.36	0.71	1.57	263	0.65	0.40	1.11	22.98

At Cancer Hospital, the annual mean dose ranged from 0.24 to 0.69 mSv, with medians from 0.18 to 0.51 mSv. The 95th percentile peaked at 1.02 mSv in 2019. The maximum recorded dose was 45.42 mSv in 2017, which was an isolated outlier. At Fuling Hospital, the annual mean dose ranged from 0.24 to 0.68 mSv, with medians from 0.18 to 0.51 mSv. The 95th percentile reached its highest value of 1.29 mSv in 2019. The maximum recorded dose was 32.13 mSv in 2023. Fuling Hospital showed greater dose variability and more frequent occurrences of maximum doses exceeding 10 mSv from 2020 onward, as illustrated by the wider interquartile ranges and higher upper whiskers in Figure 3B compared with Figure 3A.

Linear regression results for overall mean annual doses are summarized in Table 1. Overall, no significant temporal trend was observed at Cancer Hospital, whereas a borderline significant increasing trend was noted at Fuling Hospital (*p* = 0.055).

### 3.4 Dose differences by occupational category and inter-hospital comparisons within the same category

To reduce confounding from different occupational structures, we first described the dose ranking of occupational categories within each hospital, and then directly compared the same categories between the two hospitals (Table 5).

**Table 5:** Annual effective dose (mean and maximum, mSv) by hospital, year, and occupational category, 2015–2025.

Year	Hospital	Radiotherapy		Diagnostic radiology		Interventional radiology		Nuclear medicine		Scientific research		Dental radiology		Other applications	
		Mean (mSv)	Max (mSv)	Mean (mSv)	Max (mSv)	Mean (mSv)	Max (mSv)	Mean (mSv)	Max (mSv)	Mean (mSv)	Max (mSv)	Mean (mSv)	Max (mSv)	Mean (mSv)	Max (mSv)
2015	Cancer	0.22	0.95	0.25	0.35	0.24	0.3	0.4	0.95	/	/	/	/	/	/
	Fuling	0.21	0.3	0.24	0.82	0.24	0.38	0.27	0.55	/	/	0.23	0.23	/	/
2016	Cancer	0.33	1.11	0.36	1.14	0.33	0.43	0.51	1.21	/	/	/	/	0.43	0.43
	Fuling	0.38	0.46	0.33	0.49	0.49	1.78	0.39	0.54	/	/	0.33	0.33	/	/
2017	Cancer	0.97	45.42	0.45	2.21	0.28	0.51	0.32	0.87	/	/	/	/	0.72	0.72
	Fuling	0.37	0.46	0.38	0.8	0.35	2.21	0.37	0.62	/	/	0.29	0.39	/	/
2018	Cancer	0.31	14.11	0.27	5.93	0.51	3.09	0.17	0.82	0.05	0.05	/	/	0.26	0.26
	Fuling	0.17	0.18	0.18	0.91	0.38	1.21	0.22	0.37	/	/	0.15	0.18	/	/
2019	Cancer	0.47	1.55	0.55	2.44	0.59	3.08	0.55	1.62	0.42	0.74	/	/	0.46	0.46
	Fuling	0.47	1.49	0.46	1.54	0.76	3.49	0.98	2.9	/	/	0.32	0.46	/	/
2020	Cancer	0.36	0.79	0.39	0.55	0.32	0.51	0.41	0.69	/	/	/	/	0.42	0.42
	Fuling	0.41	0.54	0.52	11.23	0.48	2.02	0.47	0.7	/	/	0.49	1.05	/	/
2021	Cancer	0.05	0.05	0.08	1.64	0.47	16.39	0.09	0.47	/	/	0.05	0.05	/	/
	Fuling	0.28	0.47	0.35	2.43	0.58	17	0.31	0.62	0.21	0.38	/	/	/	/
2022	Cancer	0.28	0.57	0.26	0.48	0.27	1.67	0.32	1.13	/	/	/	/	0.2	0.2
	Fuling	0.29	0.86	0.25	1.16	0.72	8.77	0.46	2.35	/	/	0.2	0.2	/	/
2023	Cancer	0.37	19.97	0.25	3.74	0.27	1.7	0.29	0.93	/	/	/	/	0.64	1.5
	Fuling	0.55	7.87	0.64	32.13	1.15	23.81	0.36	2.33	0.48	2.53	/	/	/	/
2024	Cancer	0.37	0.95	0.53	7.12	0.33	1.23	0.44	2.63	/	/	/	/	0.86	1.9
	Fuling	0.35	0.89	0.38	2.29	0.86	13.59	0.41	0.98	0.21	0.3	/	/	/	/
2025	Cancer	0.42	0.76	0.34	1.57	0.37	1.31	0.43	1.17	/	/	/	/	0.45	0.54
	Fuling	0.37	0.69	0.37	1.33	1.1	22.98	0.46	1.41	0.31	0.31	/	/	/	/

Within each hospital, interventional radiology personnel consistently received the highest mean doses among all categories (Cancer Hospital: 0.24–0.59 mSv; Fuling Hospital: 0.24–1.15 mSv), followed by nuclear medicine staff. Diagnostic radiology and radiotherapy staff maintained relatively low and stable mean doses.

**Figure 4:**
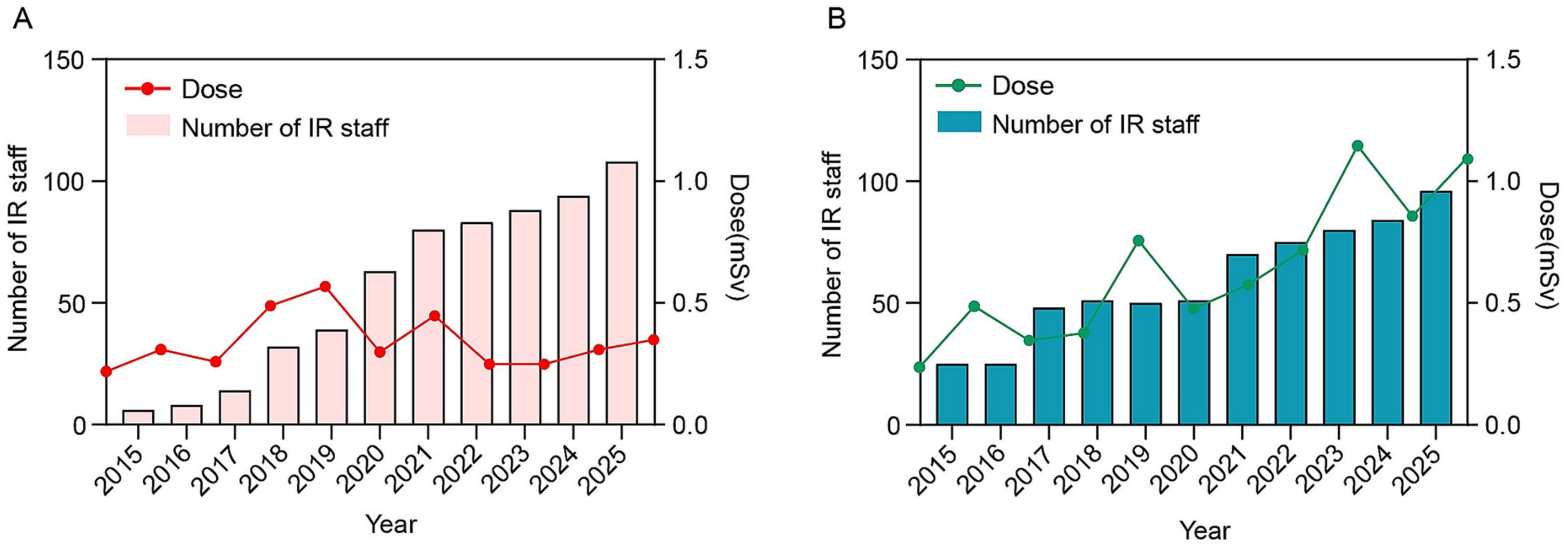
Staff number and annual effective dose among interventional radiology personnel, 2015–2025. **(A)** Annual number of IR personnel (bars) and annual mean individual effective dose (line, mSv) at Chongqing University Cancer Hospital. **(B)** Annual number of IR personnel (bars) and annual mean individual effective dose (line, mSv) at Chongqing University Fuling Hospital.

For inter-hospital comparisons of the same category, the most marked difference was observed in interventional radiology (Figures 4A,B and Table 6). At Cancer Hospital, despite a 17-fold increase in IR staff over the study period (Figure 4A, bars), the mean annual dose for this group remained stable and low (0.24–0.59 mSv; Figure 4A, line). The 95th percentile rarely exceeded 0.73 mSv, and the maximum dose never surpassed 3.1 mSv. No dose limit violations occurred. In contrast, at Fuling Hospital, IR personnel had significantly higher and more variable doses (Figure 4B). The mean dose showed an upward trend after 2019, and the 95th percentile peaked at 6.70 mSv in 2023. Maximum doses exceeded 10 mSv in multiple years and surpassed the 20 mSv limit in 2023 and 2025.

**Table 6:** Staff number, sex distribution, and annual effective dose of interventional radiology personnel in two tertiary hospitals, 2015–2025.

Year	Hospital	IR staff	Male	Female	Female (%)	Mean (mSv)	Median (mSv)	P95 (mSv)	Max (mSv)
2015	Cancer	6	4	2	33.33	0.24	0.22	0.29	0.30
	Fuling	25	17	8	33.33	0.24	0.23	0.31	0.38
2016	Cancer	8	6	2	25.00	0.33	0.32	0.42	0.43
	Fuling	25	18	7	25.00	0.49	0.37	1.27	1.78
2017	Cancer	14	12	2	14.29	0.28	0.28	0.45	0.51
	Fuling	48	32	16	14.29	0.35	0.29	1.03	2.21
2018	Cancer	32	26	6	18.75	0.51	0.40	1.14	3.09
	Fuling	51	35	16	18.75	0.38	0.36	0.81	1.21
2019	Cancer	39	30	9	23.08	0.59	0.45	1.07	3.08
	Fuling	50	37	13	23.08	0.76	0.68	1.43	3.49
2020	Cancer	63	38	25	39.68	0.32	0.35	0.46	0.51
	Fuling	51	35	16	39.68	0.48	0.44	0.69	2.02
2021	Cancer	80	46	34	42.50	0.47	0.45	0.73	1.65
	Fuling	70	48	23	42.50	0.58	0.20	1.37	17.00
2022	Cancer	83	47	36	43.37	0.27	0.26	0.38	1.67
	Fuling	75	53	22	43.37	0.72	0.29	3.03	8.77
2023	Cancer	88	49	39	44.32	0.27	0.20	0.45	1.70
	Fuling	80	55	25	44.32	1.15	0.20	6.70	23.81
2024	Cancer	94	53	41	43.62	0.33	0.29	0.67	1.23
	Fuling	84	54	30	43.62	0.86	0.44	2.24	13.59
2025	Cancer	108	57	51	47.22	0.37	0.34	0.68	1.31
	Fuling	96	61	35	47.22	1.10	0.48	2.13	22.98

For nuclear medicine, Fuling Hospital also showed higher doses (mean 0.22–0.98 mSv) compared with Cancer Hospital (mean 0.09–0.55 mSv), although the number of staff in this category was small. For radiotherapy and diagnostic radiology, mean and maximum doses were generally comparable between the two hospitals, with no consistent or substantial differences.

### 3.5 High-dose events and trends in high-risk subgroups

A total of 24 high-dose records (annual effective dose >5 mSv) were identified during the study period. These were individual annual records, with some workers possibly having repeated events in different years. The distribution of these records by hospital, year, dose range, and occupational category is presented in Table 7.

**Table 7:** Number of workers with excessive annual effective dose, 2015–2025.

Hospital	Year	Dose range (mSv)	Occupational category	Number of records
Cancer	2017	>20	Radiotherapy	1
	2018	5–10	Radiotherapy	1
	2018	10–20	Radiotherapy	1
	2021	10–20	Interventional radiology	1
	2023	10–20	Interventional radiology	1
	2024	5–10	Interventional radiology	1
Fuling	2020	5–10	Interventional radiology	1
	2021	10–20	Interventional radiology	1
	2022	5–10	Interventional radiology	3
	2023	5–10	Interventional radiology	4
	2023	10–20	Interventional radiology	1
	2023	>20	Interventional radiology	2
	2024	5–10	Interventional radiology	2
	2024	10–20	Interventional radiology	1
	2025	5–10	Interventional radiology	1
	2025	10–20	Interventional radiology	2
	2025	>20	Interventional radiology	1

At Cancer Hospital, there were 6 high-dose records across the entire period: 2 in the 5–10 mSv range and 3 in the 10–20 mSv range, including 1 record exceeding 20 mSv. These events were sporadic, with no more than one record in any single year after 2017. All high-dose records at Cancer Hospital came from radiotherapy (*n* = 3) and interventional radiology (*n* = 2) staff.

At Fuling Hospital, there were 19 high-dose records: 11 in the 5–10 mSv range, 5 in the 10–20 mSv range, and 3 exceeding 20 mSv. These events became more frequent after 2020, peaking at 7 records in 2023. The vast majority of these high-dose records (16 out of 19) were from interventional radiology personnel, with the remaining 3 from diagnostic radiology and radiotherapy staff.

Linear regression analysis for the high-risk subgroup showed that the mean annual dose for interventional radiology staff at Cancer Hospital had a non-significant decreasing trend (*β* = −0.02 mSv/year, 95% CI: −0.08 to 0.04, R^2^ = 0.12, *t* = −0.81, *p* = 0.44; Table 1). In contrast, at Fuling Hospital, the mean dose for IR staff showed a significant increasing trend over the 10-year period (β = 0.08 mSv/year, 95% CI: 0.04 to 0.12, *R*^2^ = 0.67, *t* = 4.12, *p* = 0.003; Table 1 and Figure 4B). This increasing trend was accompanied by a concentration of high-dose events among interventional radiology staff: of the 19 high-dose records at Fuling Hospital, 16 (84.2%) were from this occupational category (Table 7).

## 4 Discussion

This 10-year repeated cross-sectional study provides a comprehensive analysis of occupational radiation exposure trends among radiology staff in two tertiary hospitals in Chongqing. Our findings show that overall radiation protection in both hospitals is generally acceptable, with mean annual effective doses well below the Chinese national occupational limit of 20 mSv. However, we observed notable differences between the two institutions in workforce composition, dose stability, modality-specific exposure patterns, and the frequency of high-dose events. These differences should be interpreted with caution, as the two hospitals differ not only in geographic location (urban vs. suburban) but also in specialty type (cancer center vs. general hospital) and service mix, all of which may independently influence occupational dose profiles.

The rapid growth in the number of radiation workers reflects the ongoing expansion of radiology services in Chongqing, consistent with broader trends observed in other large Chinese cities. The urban cancer center experienced faster workforce growth and a more diversified professional structure, including substantial increases in radiotherapy, nuclear medicine, and interventional radiology. In contrast, the suburban general hospital was dominated by diagnostic and interventional radiology, with slower growth in other categories. The rising proportion of female staff in both hospitals highlights the increasing participation of women in radiation-related occupations, and underscores the need to consider sex-specific factors in occupational health protection, particularly regarding reproductive health and pregnancy policies.

Overall mean annual effective doses in both hospitals were low and comparable to those reported from other large tertiary hospitals in China and internationally. Our mean annual dose levels are comparable to those reported in other international studies. For instance, a study from Lithuania reported mean annual doses of 0.54 mSv for nuclear medicine workers (9), which is consistent with our findings. In Oman, mean annual doses for diagnostic radiology staff ranged from 0.31 to 0.86 mSv across different modalities (12). In Tanzania, radiotherapy staff received mean annual doses of 0.60 mSv, which aligns closely with our findings (15). A recent evaluation from Japan reported occupational doses of 0.15–0.45 mSv for CT healthcare workers, and a study from Turkey highlighted considerable variability in interventional cardiology staff doses and attitudes toward radiation safety (15, 16). These comparisons suggest that the dose levels observed in our two hospitals are generally within the range of international reports, although interventional radiology doses at the suburban hospital are at the higher end of the spectrum.

However, dose stability and extreme events differed substantially. The urban cancer center maintained relatively stable doses over the decade, with only sporadic high-dose events and no dose-limit violations after 2017. In contrast, the suburban general hospital exhibited greater dose fluctuations, more frequent high-dose episodes, and repeated breaches of dose limits in recent years. These differences likely reflect a combination of factors, including equipment renewal cycles, shielding infrastructure, availability of protective supplies, staffing levels, and radiation protection management capacity. However, as these variables were not directly measured in this study, these interpretations remain speculative and should be treated as hypotheses to be tested in future investigations.

When examining specific occupational categories, interventional radiology personnel consistently had the highest mean doses in both hospitals, confirming that this group remains a priority for radiation protection. This finding is consistent with previous studies showing that interventional radiologists, who work close to X-ray sources in sterile environments where additional shielding is often limited, face higher occupational exposure than staff in conventional diagnostic radiology (17–19). However, our data also revealed an important nuance: the highest maximum doses recorded in the urban cancer center came from radiotherapy staff (45.42 mSv in 2017 and 14.11 mSv in 2018), not interventional radiology. This highlights the need to monitor not only mean doses but also extreme values and outlier events across all modalities.

The like-for-like comparison of interventional radiology staff between the two hospitals revealed particularly striking differences. At the urban cancer center, despite a 17-fold expansion of the interventional radiology workforce, mean doses remained stable and low (0.24–0.59 mSv), with no dose limit violations. This suggests that the urban hospital effectively managed radiation safety during rapid service growth, possibly through standardized training, optimized workflows, consistent use of protective equipment, adequate shielding, and timely dose feedback. In contrast, at the suburban general hospital, interventional radiology staff experienced significantly higher mean doses (0.24–1.15 mSv), with a significant increasing trend over time (*β* = 0.08 mSv/year, *p* = 0.003), frequent high-dose events, and dose limit breaches in 2023 and 2025. These patterns may reflect challenges in maintaining radiation protection standards under increasing procedural volumes, potentially related to delayed equipment renewal, aging shielding, insufficient protective supplies, high workload, and less robust safety oversight. Nevertheless, these interpretations are hypothetical and require confirmation through direct measurement of these factors in future studies.

For other occupational categories, nuclear medicine staff also showed notable dose levels, particularly at the suburban hospital, where mean doses were higher than at the urban cancer center. Radiotherapy and diagnostic radiology staff maintained relatively low and stable doses in both hospitals, with no consistent or substantial inter-hospital differences. This suggests that the suburban-urban gap is not uniform across all modalities but is most pronounced in high-risk interventional procedures.

The pattern of high-dose events further supports the conclusion that interventional radiology is the primary driver of elevated occupational exposure in the suburban hospital. Of the 19 high-dose records (>5 mSv) in the suburban hospital, 16 were from interventional radiology staff. In contrast, the urban cancer center had only five high-dose records over the entire decade, with three from radiotherapy and two from interventional radiology. This distribution confirms that high-dose events are not random but are concentrated in specific high-risk modalities, and that hospital-level management practices likely play an important role in mitigating these risks.

Our study has several limitations that should be acknowledged. First, the data were derived from institutional dosimetry archives rather than individual-level longitudinal tracking, which limits our ability to assess cumulative doses or follow the same workers over time. Second, we did not collect detailed information on procedure volumes, fluoroscopy time, equipment type or age, use of personal protective devices, or specific work practices, which limits causal inference. Third, dosimeters were worn outside lead aprons at the left chest/shoulder, which may overestimate effective dose, especially among interventional radiology personnel who wear lead aprons. We also lacked under-apron, lens, and hand dose measurements. Fourth, the two hospitals differ in both location and specialty type, so the observed differences cannot be uniquely attributed to urban versus suburban settings. Fifth, only two hospitals were included, limiting the generalizability of our conclusions to other settings. Finally, some high-dose events may represent repeated records from the same individual across different years, although the repeated cross-sectional design prevents us from confirming this. In addition, variability in dosimeter wearing compliance across individuals and departments may have contributed to either underestimation or overestimation of actual doses. While dosimeters were issued and collected quarterly according to standard protocols, we could not verify the consistency of wearing practices for each worker throughout the monitoring period. This may affect the comparability of dose estimates between the two hospitals.

Despite these limitations, our findings have important implications for occupational health practice. The fact that one hospital maintained stable low doses during rapid workforce expansion suggests that effective radiation protection is achievable with proper management. The suburban hospital, where interventional radiology staff showed increasing dose trends and frequent high-dose events, would benefit from targeted interventions, including upgrading shielding systems, ensuring consistent use of personal protective equipment, standardizing interventional procedures, strengthening radiation safety training, and implementing real-time dose monitoring with early warning systems. Urban hospitals with robust safety records can serve as benchmarks for best practices.

In conclusion, this 10-year study of two tertiary hospitals in Chongqing demonstrates that overall occupational radiation exposures are within acceptable limits, but that significant inter-hospital differences exist, particularly in interventional radiology. These differences likely reflect a combination of hospital type, service mix, workload, and management practices rather than geographic location alone. Continuous, standardized personal dose monitoring remains fundamental to protecting healthcare workers exposed to radiation, and future multi-center studies with detailed procedural and equipment data are needed to further elucidate the determinants of occupational dose variability.

## 5 Conclusion

In this 10-year repeated cross-sectional study of radiation workers at two tertiary hospitals in Chongqing, annual effective doses remained generally within safe occupational limits, with overall means ranging from 0.24 to 0.69 mSv. Interventional radiology personnel consistently had the highest mean doses among all occupational categories, confirming them as the primary high-risk group for routine exposure. However, extreme maximum doses were observed in radiotherapy staff at the urban cancer center, indicating that outliers occur across multiple modalities and require attention. The two hospitals showed distinct dose stability and high-dose event patterns: the urban cancer center maintained stable low doses despite rapid workforce growth, whereas the suburban general hospital exhibited greater dose variability, a significant increasing trend in interventional radiology mean doses (*β* = 0.08 mSv/year, *p* = 0.003), and more frequent dose-limit breaches. These differences likely reflect a combination of hospital type, service mix, workload, and management practices. The findings are specific to the two institutions studied and should not be generalized to all urban or suburban hospitals without further evidence. To reduce occupational radiation risks, tailored protection strategies—including strengthened shielding, consistent use of protective equipment, standardized procedures, and real-time dose monitoring—should be prioritized, particularly in settings with less robust radiation protection infrastructure. Continuous, high-quality personal dose monitoring remains essential for safeguarding the long-term health of healthcare workers exposed to ionizing radiation.

## Data Availability

The raw data supporting the conclusions of this article will be made available by the authors, without undue reservation.
