# Supplementary material for: A 10-year retrospective repeated cross-sectional study of occupational radiation exposure trends among radiology staff in two tertiary hospitals in Chongqing
Source: Front Public Health. 2026 Aug 5;14:1888215. doi: 10.3389/fpubh.2026.1888215 (PMC13485746; doi:10.3389/fpubh.2026.1888215)
Supplement: Supplementary file 1 [file Data_Sheet_1.pdf]

Table S1. Number of radiation workers included, excluded, and total annual records in two tertiary hospitals, 2015–2025.

Year	Chongqing university cancer hospital			Chongqing university Fuling hospital		
	Included (N)	Excluded (N)	Total records	Included (N)	Excluded (N)	Total records
2015	76	0	76	100	1	101
2016	105	0	105	107	1	108
2017	156	2	158	154	0	154
2018	231	3	234	166	2	168
2019	285	3	288	168	1	169
2020	290	2	292	171	0	171
2021	392	4	396	218	2	220
2022	445	5	450	230	2	232
2023	472	5	477	248	0	248
2024	497	8	505	258	1	259
2025	536	7	543	263	2	265
